# Gene Therapy for Neuropathic Pain by Silencing of TNF-α Expression with Lentiviral Vectors Targeting the Dorsal Root Ganglion in Mice

**DOI:** 10.1371/journal.pone.0092073

**Published:** 2014-03-18

**Authors:** Nobuhiro Ogawa, Hiromichi Kawai, Tomoya Terashima, Hideto Kojima, Kazuhiro Oka, Lawrence Chan, Hiroshi Maegawa

**Affiliations:** 1 Department of Medicine, Molecular Genetics in Medicine, Shiga University of Medical Science, Otsu, Shiga, Japan; 2 Department of Molecular Genetics in Medicine, Shiga University of Medical Science, Otsu, Shiga, Japan; 3 Departments of Medicine and Molecular and Cellular Biology, Baylor College of Medicine, Houston, Texas, United States of America; Hertie Institute for Clinical Brain Research, University of Tuebingen, Germany

## Abstract

Neuropathic pain can be a debilitating condition. Many types of drugs that have been used to treat neuropathic pain have only limited efficacy. Recent studies indicate that pro-inflammatory mediators including tumor necrosis factor α (TNF-α) are involved in the pathogenesis of neuropathic pain. In the present study, we engineered a gene therapy strategy to relieve neuropathic pain by silencing TNF-α expression in the dorsal root ganglion (DRG) using lentiviral vectors expressing TNF short hairpin RNA1-4 (LV-TNF-shRNA1-4) in mice. First, based on its efficacy in silencing TNF-α *in vitro*, we selected shRNA3 to construct LV-TNF-shRNA3 for *in vivo* study. We used L5 spinal nerve transection (SNT) mice as a neuropathic pain model. These animals were found to display up-regulated mRNA expression of activating transcription factor 3 (ATF3) and neuropeptide Y (NPY), injury markers, and interleukin (IL)-6, an inflammatory cytokine in the ipsilateral L5 DRG. Injection of LV-TNF-shRNA3 onto the proximal transected site suppressed significantly the mRNA levels of ATF3, NPY and IL-6, reduced mechanical allodynia and neuronal cell death of DRG neurons. These results suggest that lentiviral-mediated silencing of TNF-α in DRG relieves neuropathic pain and reduces neuronal cell death, and may constitute a novel therapeutic option for neuropathic pain.

## Introduction

Neuropathic pain is defined as pain caused by a lesion or disease in the somatosensory nervous system [1]. Its severity varies but is often so debilitating that it has been called “the most terrible of all tortures, which a nerve wound may inflict” [2]. Neuropathic pain is characterized by sensory abnormalities that range from unpleasant abnormal sensations (dysesthesia), to an increased response to painful stimuli (hyperalgesia), and to pain in response to a stimulus that does not normally provoke pain (allodynia) [3]. These three kinds of pain are the most difficult types of chronic pain to treat, and are often associated with autoimmune diseases such as rheumatoid arthritis or Sjögren's syndrome, metabolic diseases such as diabetes mellitus, side effects of drugs for cancer or HIV chemotherapy, toxin exposure, infection and trauma [4]–[7]. Neuropathic pain impairs a patient's daily activities and quality of life. Currently available treatments for neuropathic pain include sodium channel blockers, anti-depressants and anti-epileptic drugs, but they are grossly inadequate; novel treatment methods with better efficacy are much needed.

A number of animal models have been developed that can be used to study the mechanism of neuropathic pain and to examine the efficacy of new therapies [8]. For example, L5 spinal nerve injury mouse models are widely used, so is the modified spinal nerve ligation model [9], in which severe mechanical allodynia and hyperesthesia occur without motor deficit. Another model, the spinal nerve transection (SNT) mice [10] requires skillful surgery; these mice display a stable type of neuropathic pain and is especially suitable for pain studies and behavioral tests.

Recently, peripheral nerve and spinal nerve injury models have also been used for research into the pathogenesis of neuropathic pain. Studies in these models revealed that pro-inflammatory mediators, such as tumor necrosis factor α (TNF-α), interleukin (IL)-1β and IL-6, are up-regulated in the dorsal root ganglion (DRG) and may be important mediators of neuropathic pain in rodents [7], [11]–[15]. Of note, TNF-α signaling was shown to stimulate sensory neuronal excitability [16] and produce neuropathic pain [17], [18]. TNF-α and caspase-related signaling pathways also result in cell death and neuropathic pain [19], establishing TNF-α expression in the DRG as a key mediator of neuropathic pain. In addition, we previously reported that TNF-α was involved in the pathogenesis of diabetic neuropathy in mice and that inactivation or inhibition of TNF-α ameliorated diabetic neuropathy and reduced hyperalgesia [20], [21]. In nondiabetic injury neuropathic pain models, the inhibition of TNF-α also reduced neuropathic pain-related behavior [13], [22]–[24]. Furthermore, in human studies, TNF-α was found to be up-regulated in sensory nerve and DRG, and contributed to neuropathic pain in sensory neuronopathy, inflammatory diseases or nerve injury [7], [25]–[27]. We therefore hypothesized that TNF-α may be a suitable therapeutic target for the treatment of neuropathic pain and neuropathy. Indeed, human studies have suggested that anti-TNF-α drugs ameliorate neuropathic pain and neuropathy in Sjögren's syndrome [25], sciatica and disc-herniation [28]–[30]. However, the need for these drugs to be delivered systemically led to serious side effects such as infection, interstitial pneumonia and liver failure because generalized TNF-α inhibition suppresses the normal immune system. Thus, to avoid inducing systemic side effects, we selected the local administration of gene delivery vectors to target the TNF-α at the site of injection.

Here, we present a gene therapy strategy that relieves SNT-induced neuropathic pain by silencing TNF-α expression in DRG using RNA interference technology with lentiviral vectors. This strategy was effective in suppressing neuropathic pain and protecting DRG neurons from cell death. These results constitute a proof of principle that the therapeutic strategy can potentially be used to treat neuropathic pain and sensory neuronopathy in people.

## Materials and Methods

### Ethics statement

All animal protocols were approved by the Institutional Animal Care and Use Committee of Shiga University of Medical Science (Approved Number: 2012-8-5). All procedures were performed in accordance with the guidelines of the Research Center for Animal Life Science of Shiga University of Medical Science.

### Construction of mouse TNF-α over-expression or silencing plasmids for lentiviral vectors

The full-length mouse TNF-α cDNA was inserted into pLL3.7 plasmid including elongation factor 1 (EF-1) promoter and DsRed as a reporter gene (TNF-α over-expression plasmid: LV-TNF-DsRed). Four short hairpin RNAs (shRNA) were individually inserted into pLL3.7 plasmid including U6 promoter and enhanced green fluorescent protein (GFP), as a reporter gene, driven by cytomegalovirus (CMV) promoter (TNF-α silencing plasmid: LV-TNF-shRNA1-4). The full-length TNF-α coding sequence (NM_013693) was isolated from RAW cells with lipopolysaccharide stimulation. shRNA sequences against mouse TNF-α were designed using a web site from The RNAi Consortium (TRC) algorithm (Available: http://www.broadinstitute.org/rnai/trc. Accessed on 2014 Feb 20.).

### Production of mouse TNF-α over-expressing or silencing lentiviral vectors

To produce recombinant lentiviral particles, psPAX2 (a packaging plasmid), pMD2.G (an envelope plasmid, which has the vesiculo-stomatitis virus G-protein), a gift of Dr. Didier Trono (École Polytechnique Fédérale de Lausanne, Switzerland), and TNF-α over-expression plasmid (or silencing plasmid) were co-transfected into 293T cells using the calcium phosphate method as described previously [31]. After 6 h incubation, the culture medium was exchanged. Culture medium containing lentiviral particles was collected after 48 h incubation, and was filtered through a 0.45 μm pore size cellulose acetate filter. Lentiviral vectors were concentrated using Lenti-X Concentrator (Takara Bio Inc., Otsu, Japan), and were stocked at a concentration of 1.8×10^8^ infectious units (IFU)/ml at −80°C after the determination of IFU using 293T cells. Finally, we produced LV-TNF-DsRed (mouse TNF-α and DsRed expression vector), LV-GFP (control vector expressing GFP without shRNA sequence) and LV-TNF-shRNA1-4 (TNF-α silencing vector with GFP expression).

### Co-infection test of mouse TNF-α over-expressing and silencing lentiviral vectors

One day before infection, 293T cells were split to 6 well-culture dishes at a density of 1.0×10^5^ cells per well. The confluency of 293T cells was approximately 70% at the time of infection. At 6 h after infection of LV-TNF-DsRed (3.6×10^5^ IFU), 3.6×10^5^ IFU LV-GFP or LV-TNF-shRNA1-4 was separately added into each well of LV-TNF-DsRed-infected 293T cells. At 72 h later, DsRed and GFP expression were observed under fluorescence microscopy and TNF-α mRNA expression was determined by quantitative RT-PCR analysis.

### Animals

Male 9–10 week-old C57BL6 (Japan CLEA, Osaka, Japan) mice weighing 19.0–22.0 g were used in this study. The mice were housed in separated cages and the room kept under 12:12 light-dark cycle with free access to food and water.

### Surgical procedures

**Figure 1:**
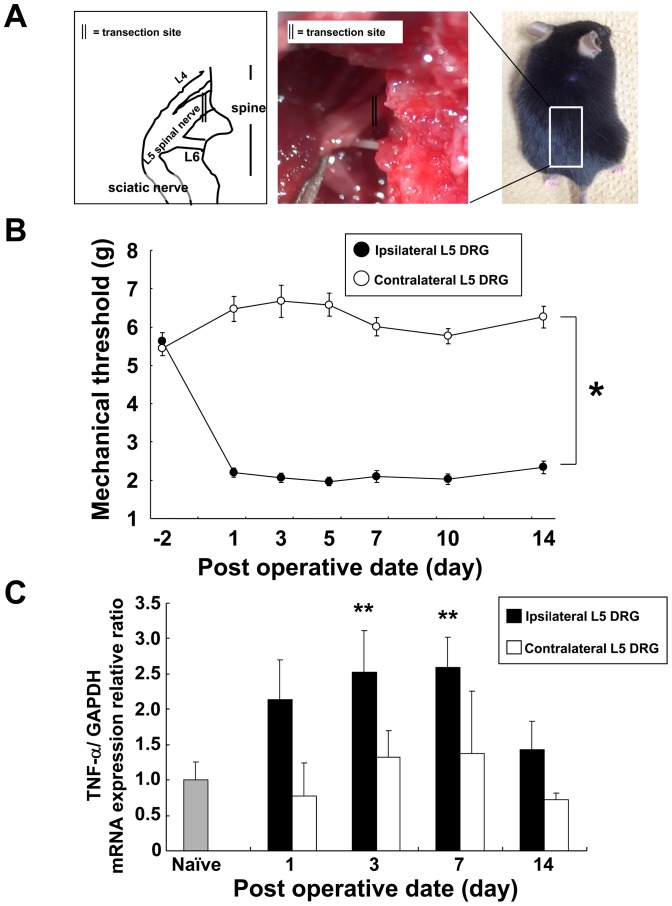
L5 spinal nerve transection as a neuropathic pain model. (**A**) Schematic presentation of left L5 spinal nerve transection (SNT) surgical procedure. Left and middle panels show the anatomical enlargement scene of the same field as the square of the mouse back in the right panel. The left panel shows a schematic view of the middle panel picture. The double lines show the L5 spinal nerve transection site in the left and middle panels. (**B**) The threshold of mechanical allodynia in the neuropathic model mice induced by SNT for 14 days. Black circles indicate ipsilateral and white circles indicate contralateral responses for mechanical stimuli. The threshold values were measured for five mice at each time point. Data show means ± S.E. *p<0.01. (**C**) TNF-α mRNA expression in bilateral L5 DRG of neuropathic pain model mice for 14 days after SNT. The data are normalized to GAPDH and presented as the relative ratio to naïve DRG (no SNT mice) (n = 3–5 per each time course group). Gray bar is naïve DRG group, black bars are ipsilateral L5 DRG group, and open bars are contralateral L5 DRG group. Bars show means ± S.E. **p<0.05, compared with naïve.

The neuropathic pain model by L5 spinal nerve transection (SNT) was generated by the method described previously (**Figure 1A**) [9], [10]. Briefly, mice were anesthetized by intraperitoneal administration of sodium pentobarbital (5 mg/kg). After the midline incision of mouse back skin, the bilateral L5 transverse processes of the lumbar spine were clearly removed. Bilateral L5 spinal nerves were exposed on the visual field. Only the left nerve was pinched and transected. Immediately after transection, 1 μl (1.8×10^5^ IFU/μl) of lentiviral vector was injected onto proximal transected site of left L5 spinal nerve using a Hamilton syringe (8001 1701LT, Hamilton company, Nevada, USA) with a 30G needle. The right spinal nerve, as a control side, was clearly exposed without transection or administration of lentiviral vector.

### Behavioral test

Mechanical allodynia was assessed prior to injury (day -2) and at 1, 3, 5, 7, 10 and 14 days after SNT, using a dynamic planter aesthesiometer (Ugo Basil, Varese, Italy) as described previously [32]. Paw withdrawal in response to mechanical stimuli was measured. Briefly, each mouse was placed in a polypropylene box with a metallic mesh floor and allowed to acclimatize to the testing environment for at least 1 h. A stimulating filament probe was positioned under the hind paw, which was gradually applied until mice withdrew their paw. The pressure was increased at approximately 10 g/mm^2^/sec. The test was performed on both the intact right or neuropathic left hind paw. The withdrawal threshold was determined by mean of three trials.

### Reverse transcription-PCR and quantitative RT-PCR analysis

Animal tissues were removed under deep anesthesia and immediately frozen in liquid nitrogen. Total RNA was extracted from cultured cells and frozen tissues using RNeasy mini kit (Qiagen, Valencia, CA) with DNase I (Rnase-free DNase set, Qiagen) treatment. Reverse transcription was performed from 100 ng of total RNA in each tube using Prime Script perfect Real time (Takara Bio Inc.).

RT-PCR was performed with the following primers: 5′-CACGTCGTAGCAAACCACCAAGTGG-3′ and 5′-GATAGCAAATCGGCTGACGGTGTGG-3′ for mouse TNF-α, 5′-AGGACGACGGCAACTACAAGAC-3′ and 5′-GAAGTTCACCTTGATGCCGTTC-3′ for GFP, 5′-AAATCCCATCACCATCTTCCA-3′ and 5′-AATGAGCCCCAGCCTTCTC-3′ for human GAPDH (for 293T cell), 5′-AACGACCCCTTCATTGAC-3′ and 5′-TCCACGACATACTCAGCAC-3′ for mouse GAPDH (for mouse tissues). For RT-PCR by TaqMan method, the following primers were used: Mn_99999915_g1 (Invitrogen, Carlsbad, USA) for mouse GAPDH and 5′-CATCTTCTCAAAATTCGAGTGACAA-3′, 5′-TCGGAGTAGACAAGGTAGAACCC-3′ and 5′-FAM-GCACGTCGTAGCAAACCACCACCAAGTGGA-TAMRA-3′ (probe) for mouse TNF-α. The PCR reaction consisted of denaturation at 94°C for 30 seconds, annealing at 60°C for 30 seconds, extension at 72°C for 30 seconds. The PCR products were analyzed with FAS-IV (Japan Genetics, Tokyo, Japan).

For quantification of each gene, real time PCR assay was performed using a LightCycler 480 (Roche Diagnostics, Manheim, Germany) with SYBR Green method (for activating transcription factor 3 (ATF3), calcitonin gene related peptide (CGRP), IL-6, neuropeptide Y (NPY), human GAPDH, mouse GAPDH and mouse TNF-α) and TaqMan method (for mouse GAPDH and mouse TNF-α). The PCR parameters were one cycle of 95°C for three min, followed by 50 cycles of denaturation at 95°C for 30 sec, annealing at 60°C for 30 sec and extension at 72°C for 30 sec. The emitted fluorescence for each reaction was measured three times during the annealing extension phase, and amplification plots were analyzed using LightCycler 480 software, version 1.5 (Roche Diagnostics Manheim, Germany). Potential genomic DNA contamination was controlled by the use of intron-encompassing primers and DNase digestion. GAPDH and MAPK6 have been reported to be the most stable reference genes for use in normalizing transcript level of interest genes although the expression of other many house-keeping genes reduced on the early phase in DRG after nerve injury [33]. Therefore, the normalization and relative expression analysis of target genes were performed using the 2^−ΔΔCt^ method with GAPDH as a control, which expression levels were confirmed not to be changed among day -2, 3, 7 and 14.

### Immunohistochemistry

Animals were anesthetized by intraperitoneal administration of sodium pentobarbital (50 mg/kg) and intracardially perfused with 30 ml of PBS followed by a fixative containing 4% paraformaldehyde in 0.1 M PB. After perfusion fixation, animal tissues were kept in the same fixative at 4°C for 18 h and permeated with 15% (w/v) sucrose buffer with agitation. Each tissue was embedded in Tissue-Tek O.C.T compound (Sakura, Tokyo, Japan), frozen with liquid nitrogen and cut on a cryostat at 8 μm sections collected on MAS (Matsunami aggressive silane)-coated glass slides. Sections were blocked with 3% normal goat serum in PBS at room temperature for 15 min and processed for immunohistochemistry. The following antibodies were used: anti-TNF-α (1∶100, Abcam, Cambridge, United Kingdom), anti-GFP (1∶100, Medical & Biological Laboratories, Nagoya, Japan), anti-cleaved caspase3 (1∶100, Cell Signaling Technology Japan, Tokyo, Japan), and AlexaFluor 488, 594 and 633 (1∶1000, Abcam) as primary and secondary antibodies. For marker stains, we used 4′,6-diamidino-2-phenylindole (DAPI) for nuclei and fluorescent Nissl (NeuroTrace 435/455 or 530/615, Molecular Probes, Eugene, Oregon, USA) for neurons. Fluorescence images were captured and analyzed using Nikon C1si and the software, EZ-C1 version 3.90 (Nikon, Tokyo Japan).

### DRG neuron cell count

To evaluate the neuronal cell survival in ipsilateral L5 DRG after SNT, we perfused mice transcardially with 4% paraformaldehyde in 0.1 M PBS, and isolated DRG tissues and embedded them in paraffin. L5 DRG paraffin blocks were sectioned at 10 μm thickness and stained with 0.1% Cresyl violet (Nissl stain, Muto Pure Chemicals Co. Ltd, Tokyo, Japan). After Nissl staining (Cresyl violet), all visible neurons in each left L5 DRG were counted in consecutive sections (20 μm intervals). For the evaluation of apoptosis after SNT with or without gene therapy, we counted the number of cleaved caspase3 positive neurons. Approximately 300 neurons were analyzed in consecutive sections (30 μm intervals) for each left L5 DRG.

Transduction efficacy and treatment effects of gene therapy by lentiviral vector *in vivo* were evaluated by counting the number of GFP or TNF-α positive neurons with the neuronal marker stain of fluorescent Nissl (NeuroTrace 435/455 for GFP or NeuroTrace 530/615 for TNF-α) in DRG after SNT with or without gene therapy. We analyzed approximately 300 neurons in consecutive sections (30 μm intervals) for each L5 DRG.

### Statistical analysis

Statistical analysis was performed with SPSS 17.0 software (SPSS Inc., Chicago, USA). All data are shown as mean ± standard error of the mean (S.E.). The one-way ANOVA followed by Tukey's test was used to calculate statistical significance for multiple data sets. For behavioral test, two-way ANOVA was used. Data were considered significant difference at *p*<0.05.

## Results

### TNF-α expression and behavior testing in a spinal nerve transection (SNT) mouse model of neuropathic pain

We induced neuropathic pain in mice by SNT on the left side of the L5 spinal nerve at a location close to the spine (ipsilateral side, **Figure 1A**). The right L5 spinal nerve was not transected (contralateral side). SNT induced severe mechanical allodynia and hyperesthesia in the ipsilateral hind paw. We measured the hind paw withdrawal threshold against the mechanical stimuli (mechanical threshold) for the ipsilateral and contralateral paws for 14 days after SNT and found that the threshold was markedly decreased the day after SNT and remained at the same low level for 14 days (**Figure 1B**). We quantified TNF-α mRNA expression in L5 DRG by RT-PCR on day 1, 3, 7 and 14 after left SNT-induced neuropathic pain (**Figure 1C**) and found that SNT led to elevation of TNF-α mRNA expression in the DRG on the ipsilateral side, which was approximately 2.5-fold higher than the level in the naïve L5 DRG on day 3 and 7 after SNT, and returned towards baseline levels on day 14 after SNT (**Figure 1C**).

### Design and evaluation of shRNA sequences against TNF-α expression in cultured cells

**Figure 2:**
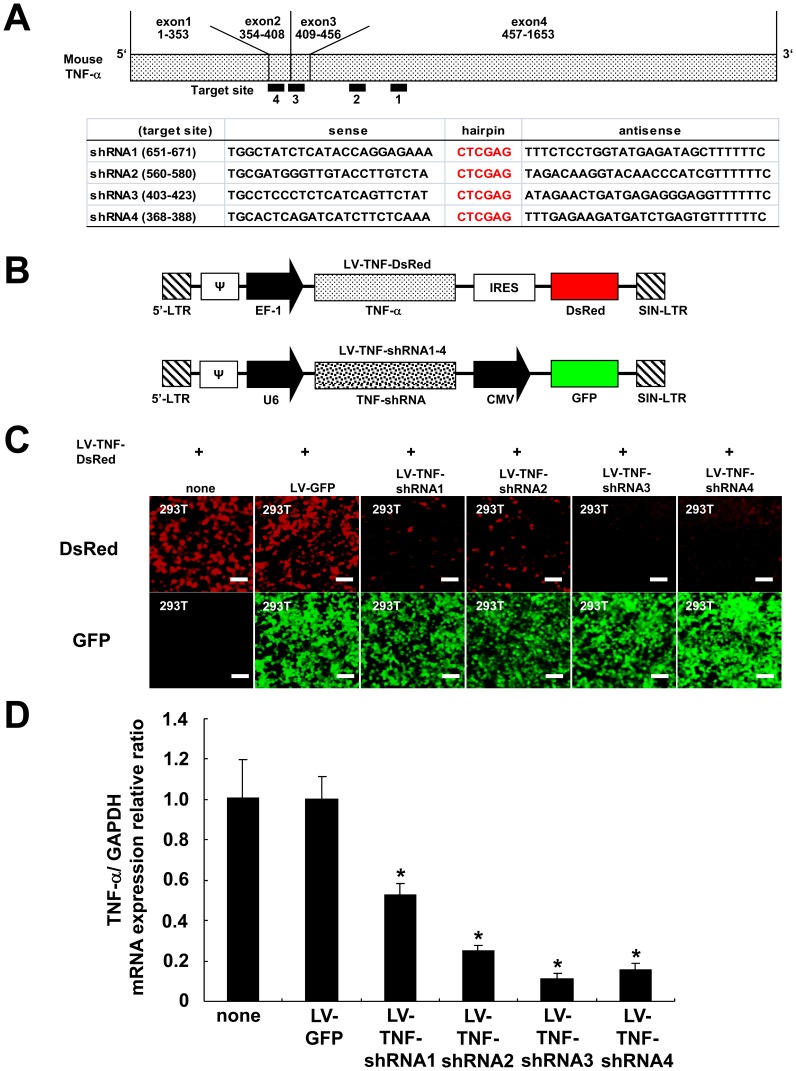
Construction of mouse TNF-α over-expression and shRNA lentiviral vectors and silencing efficacy of LV-TNF-shRNAs. (**A**) Four candidate shRNA sequences against TNF-α (shRNA1 to shRNA4) and schematic presentation of target site of these shRNAs on mouse TNF-α sequence. The black bars and the number 1 to 4 under the scheme of mouse TNF-α cDNA indicate the location of mouse TNF-α cDNA targeted by shRNA1-4. The numbers in the parentheses beside shRNA1-4 mean the location of target site against the mouse TNF-α cDNA. (**B**) Schematic presentation of LV-TNF-DsRed (TNF-α over-expression vector, upper) and LV-TNF-shRNA1-4 (TNF-α silencing vector, lower). LTR: long terminal repeat, **Ψ**: packaging signal, EF-1: elongation factor 1 promoter, IRES: internal ribosome entry sites, SIN-LTR: self inactivating-LTR, CMV: cytomegalovirus promoter. (**C**) Fluorescence in 293T cells at 72 h after co-infection with two lentiviral vectors, LV-TNF-DsRed and LV-TNF-shRNA1-4, LV-GFP (GFP control vector with no TNF-shRNA sequence) or none. Upper panels show TNF-α expression (red), lower panels show each transduction of LV-TNF-shRNA1-4 therapeutic vectors or LV-GFP (green). Scale bar  = 50 μm. (**D**) TNF-α mRNA expression in 293T cells at 72 h after infection of LV-TNF-DsRed with LV-TNF-shRNA1-4, LV-GFP, or none (n = 5 per group). The data were normalized by GAPDH. Bars indicate means ± S.E. *p<0.01 compared with LV-GFP group.

To develop an shRNA gene therapy against TNF-α in the SNT model, we designed 4 candidate shRNA sequences against TNF-α based on scoring algorithms presented by The RNAi Consortium (TRC, available: http://www.broadinstitute.org/rnai/trc. Accessed on 2014 Feb 20.) (Target sites of these four shRNA against mouse TNF-α are shown in **Figure 2A**). We cloned the individual shRNA sequences (shRNA1-4 in **Figure 2A**) into a pLL3.7 plasmid to produce lentiviral vectors designated LV-TNF-shRNA1-4 (**Figure 2B**). In these vectors, the shRNA sequence was driven by the U6 promoter and the GFP was driven by the CMV promoter (**Figure 2B**).

We created TNF-α-expressing 293T cells by infecting them with lentiviral vector over-expressing TNF-α and DsRed driven by EF-1 promoter (LV-TNF-DsRed in **Figure 2B**). We next tested the capacity of LV-TNF-shRNA1-4 to silence TNF-α expression by infecting these cells with LV-TNF-shRNA1-4 or LV-GFP (control GFP vector with no shRNA sequence). Seventy-two hours later, we observed these cells under the fluorescence microscope. We found that in the LV-TNF-shRNA1-4 and LV-GFP groups, the GFP signals were similar in 293T cells (lower panels in **Figure 2C**) confirming approximately similar level of infection of the 4 silencing vectors. In cells that were infected with silencing vectors (LV-TNF-shRNA1-4), the DsRed signal was markedly attenuated in all 4 groups compared with the LV-GFP group or single infection LV-TNF-DsRed (‘none’ group) (upper panels in **Figure 2C**). Next, we quantified TNF-α mRNA expression by RT-PCR and found that the level of TNF-α mRNA was significantly reduced in the 4 LV-TNF-shRNA groups compared with LV-GFP control group (n = 5, *p*<0.01, the relative percent lowering in mRNA level were as follows: 47.8% by LV-TNF-shRNA1, 75.1% by LV-TNF-shRNA2, 89.1% by LV-TNF-shRNA3, and 84.7% by LV-TNF-shRNA4; **Figure 2D**). Thus, of the 4 vectors that we analyzed, we found LV-TNF-shRNA3 to be the most efficacious in silencing TNF-α expression in 293T cells. We therefore tested the efficacy of using LV-TNF-shRNA3 therapy on neuropathic pain in the SNT mouse model.

### Evaluation of transduction efficiency with LV-TNF-shRNA3 injection onto left L5 spinal nerve

**Figure 3:**
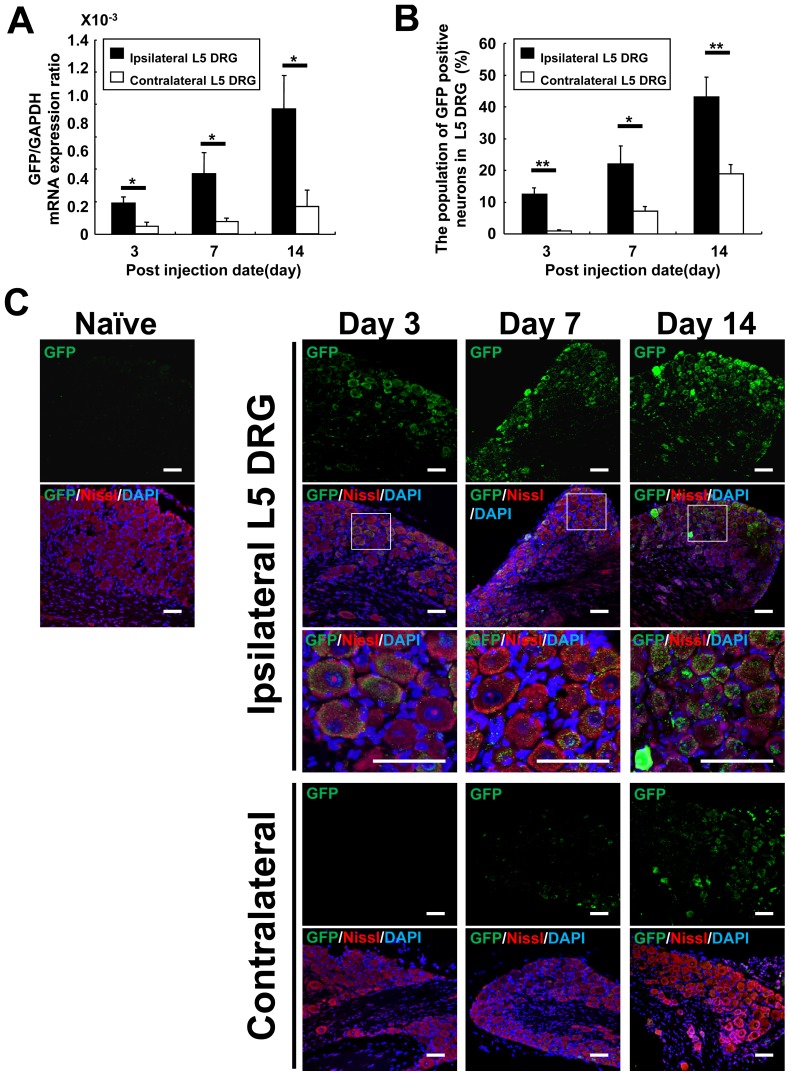
Transduction efficacy of LV-TNF-shRNA3 onto left L5 spinal nerve in L5 DRG tissues. (**A**) The quantification of GFP mRNA expression induced by LV-shRNA3 in bilateral L5 DRG on day 3, 7, 14 after SNT and injection of LV-TNF-shRNA3. The data were normalized to GAPDH. Black bars showed the results in ipsilateral L5 DRG (n = 5 per time point) and open bars showed the results in contralateral L5 DRG (n = 5 per time point). Bars indicate means ± S.E. *p<0.05. (**B**) The population of GFP positive neurons in L5 DRG on day 3, 7 and 14 after SNT and injection of LV-TNF-shRNA3. Black bars showed the results in ipsilateral L5 DRG (n = 5 per time point) and open bars showed the results in contralateral L5 DRG (n = 5 per time point). Bars indicate means ± S.E. *p<0.05, **p<0.01. (**C**) Immunohistochemical staining with anti-GFP antibodies (green), DAPI (blue) and Nissl (red) in the bilateral L5 DRG on day 3, 7 and 14 after SNT and injection of LV-TNF-shRNA3. Upper panels of each group showed immunostainigs of anti-GFP antibodies and 2nd lines' panels showed the merged images of GFP, Nissl and DAPI in each group. The square regions of 2nd lines' panels were enlarged in third lines' panels in ipsilateral L5 DRG group. Scale bar  = 50 μm.

We determined the efficiency of gene delivery after the treatment by histological analysis and mRNA expression of GFP (reporter gene) in bilateral DRG tissues after infection of LV-TNF-shRNA3 targeting the transected left L5 spinal nerve. Infection-induced GFP mRNA expression in ipsilateral L5 DRG was confirmed between day 3 to day 14 after local injection onto the left L5 spinal nerve (**Figure 3A**). mRNA expression was also observed in the contralateral L5 DRG, but the expression levels were much lower compared with the ipsilateral side. GFP mRNA expression in ipsilateral L5 DRG gradually increased from day 3 to day 14, which was also observed in the contralateral side although the expression was significantly lower (complete set of data from all treated mice, corrected to GAPDH expression, **Figure 3A**). In addition, GFP protein signals were detected mainly in neurons of L5 ipsilateral DRG sections, which population gradually increased and reached 43.2%±6.1% on day 14 (**Figure 3B, C**). In L5 contralateral DRG, GFP protein signals were observed at 1 level significantly lower than in the ipsilateral DRG, reaching 18.9%±3.0% of the neurons on day 14 (**Figure 3B, C**). Therefore, administration of LV-TNF-shRNA3 induced high level of transgene expression in DRG tissues by local injection onto the spinal nerve.

### LV-TNF-RNA3 suppressed TNF-α expression in ipsilateral L5 DRG after SNT

**Figure 4:**
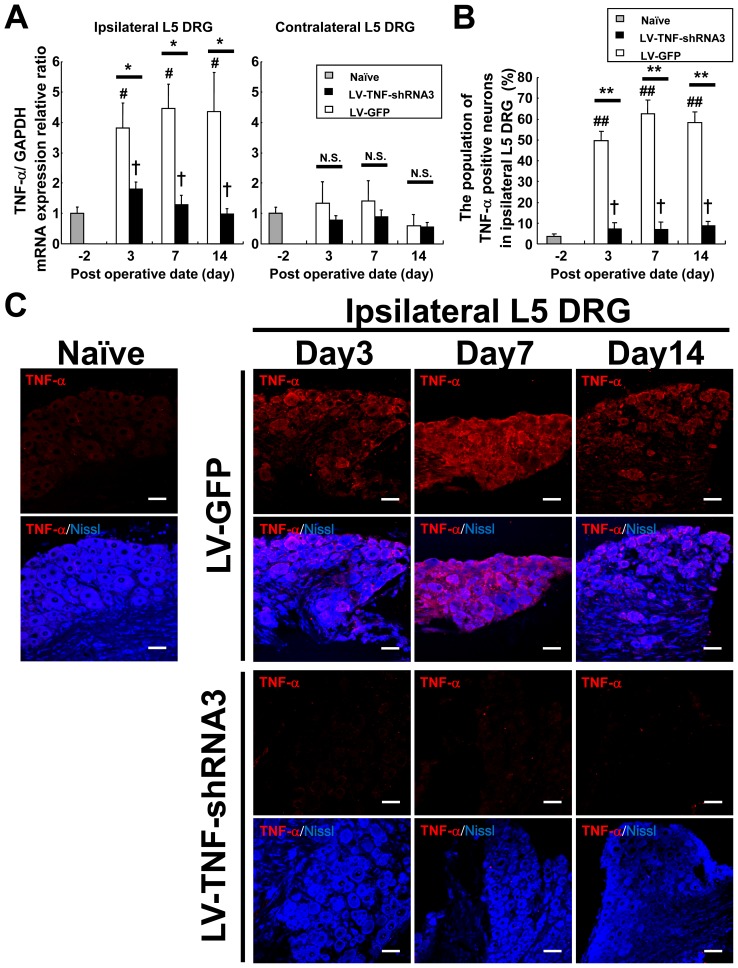
Suppression of TNF-α mRNA and protein expression in L5 DRG of SNT mice by LV-TNF-shRNA3. (**A**) TNF-α mRNA expression in bilateral L5 DRG from LV-GFP or LV-TNF-shRNA3 groups on day 3, 7, 14 after SNT and injection. Ipsilateral L5 DRG (left panel) and contralateral L5 DRG (right panel). The data are normalized to GAPDH and presented as a relative ratio to naïve DRG (on day −2). Gray bars showed the result in naïve DRG (on day −2, n = 6), white bars showed the results in LV-GFP group (n = 6–8 per time point) and black bars showed the results in LV-TNF-shRNA3 group (n = 6–8 per time point) Bars indicate means ± S.E. *p<0.05, ^#^ *p*<0.05 compared with naïve, ^##^ *p*<0.01 compared with naïve, † the difference was not significant compared with naïve. N.S. means “not significant.” (**B**) The population of TNF-α positive neurons in ipsilateral L5 DRG on day 3, 7 and 14 after SNT and injection of LV-GFP or LV-TNF-shRNA3. Gray bars showed the result in naïve DRG without SNT and injection (on day −2, n = 4), white bars showed the results in LV-GFP group (n = 4–5 per time point) and black bars showed the results in LV-TNF-shRNA3 group (n = 4–5 per time point) Bars indicate means ± S.E. ** *p*<0.01, ^##^ *p*<0.01 compared with naïve, † the difference was not significant compared with naïve. (**C**) Immunohistochemical staining with anti-TNF-α antibodies (red) and Nissl (blue) in the ipsilateral L5 DRG on day 3, 7 and 14 after SNT and injection of LV-GFP or LV-TNF-shRNA3. Upper panels of each group showed immunostainigs of anti-TNF-α antibodies and lower panels showed the merged image of TNF-α and Nissl in each group. Scale bar  = 50 μm

We quantified the relative degree of TNF-α silencing after LV-TNF-shRNA3 gene therapy by quantitative RT-PCR of TNF-α mRNA and immunohistochemistry of TNF-α protein in L5 DRG tissues. In SNT with LV-GFP group (control group), TNF-α mRNA expression increased approximately 4-fold compared with pretreatment expression levels in ipsilateral L5 DRG from day 3 to day 14 (left panel in **Figure 4A**). The increased TNF-α mRNA expression by SNT was markedly suppressed down to baseline levels in ipsilateral L5 DRG treated with LV-TNF-shRNA3 (therapy group) for 14 days (left panel in **Figure 4A**). In the contralateral L5 DRG, there was no significant change in TNF-α mRNA levels in the untreated LV-GFP groups (control group). The LV-TNF-shRNA3 group also did not show any change (right panel in **Figure 4A**). Immunohistochemistry in ipsilateral L5 DRG revealed markedly increased immunoreactive TNF-α in the cytosol of neurons after ipsilateral SNT in the LV-GFP group compared with naïve (control group) from day 3 to day 14 (**Figure 4B, C**). The immunostained TNF-α positive neurons (co-localized with Nissl stain) increased markedly in SNT with LV-GFP group compared with pretreatment DRG group (naïve) from day 3 to day 14 (the percentage of positive neuron were increased approximately 60% and peaked at day 7, **Figure 4B**). In gene therapy group with LV-TNF-shRNA3, the appearance of immunoreactive TNF-α-positive neurons induced in the vast majority of the DRG neurons by SNT was suppressed to baseline levels from day 3 to day 14 (**Figure 4B, C**). These results were consistent with the data on the level of TNF-α mRNA expression.

### LV-TNF-RNA3 preserves DRG neurons from cell death caused by SNT

**Figure 5:**
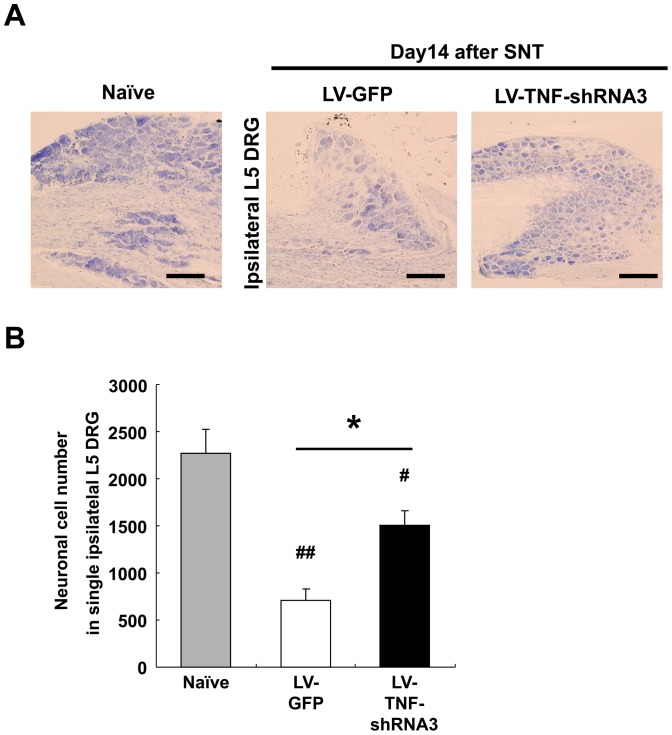
Evaluation of survival neurons in L5 DRG after SNT with LV-TNF-shRNA3. (**A**) Nissl stain of L5 ipsilateral L5 DRG on day 14 after SNT with LV-TNF-shRNA3 or LV-GFP. Scale bar  = 50 μm. (**B**) Quantitative analysis of cell survival in L5 DRG neurons after SNT with LV-TNF-shRNA3 or LV-GFP (n = 5 per group). Gray bar - neuronal cell numbers including single ipsilateral L5 DRG in naïve DRG group; white bar - LV-GFP group; black bar - LV-TNF-shRNA3 group. Bars show means ± S.E. *p<0.01, ^#^ *p*<0.05 compared with naïve, ^##^ *p*<0.01 compared with naïve.

SNT induced cell death leading to reduced neuron number was reported to correlate with neuropathic pain [34]. We therefore used Nissl staining to quantify the number of surviving DRG neurons in ipsilateral L5 DRG in LV-GFP and LV-TNF-shRNA3 treatment groups on day 14 after SNT (**Figure 5A, B**). Neuronal cell numbers were significantly reduced by SNT in the ipsilateral L5 DRG in LV-GFP group (709.8±119.1 cells/DRG tissue) compared to naïve (2264±260.3 cells/DRG tissue) (**Figure 5A, B**). In contrast, DRG neuronal cell numbers were significantly preserved in the LV-TNF-shRNA3 treatment group (1501±154.8 cells/DRG tissue) compared with the LV-GFP group (**Figure 5A, B**).

**Figure 6:**
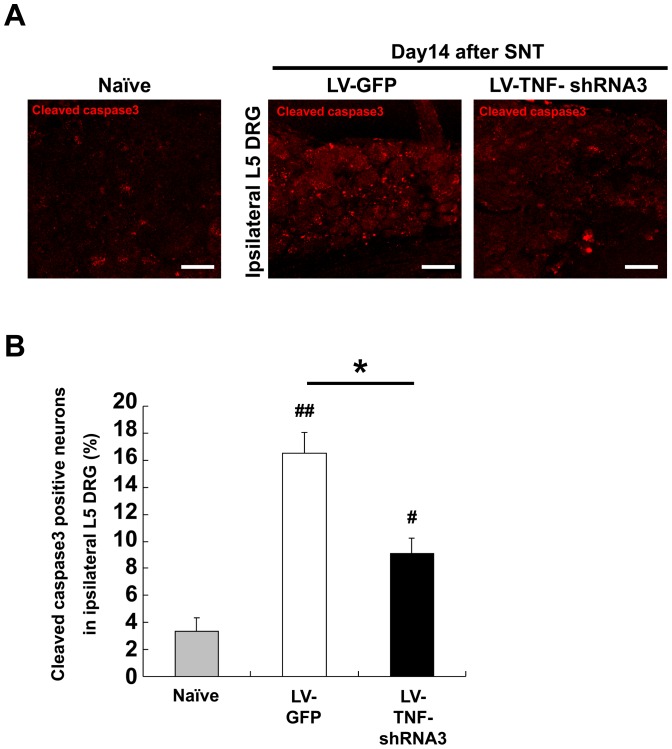
Immunohistochemical analysis of cleaved caspase3 in DRG tissues. Immunohistochemical staining of cleaved caspase3 (red) (**A**) and the quantitative analysis of cleaved caspase3 positive neurons (**B**) in ipsilateral L5 DRG after spinal nerve transection with LV-TNF-shRNA3 or LV-GFP on day 14 (n = 5 in each group). Bars show means ± S.E. *p<0.01, ^#^ *p*<0.05 compared with naïve, ^##^ *p*<0.01 compared with naïve. Scale bar  = 50 μm.

We next used immunohistochemistry of cleaved caspase3 to quantify the number of apoptotic neurons in L5 ipsilateral DRG on day 14 after SNT and the therapeutic effects of TNF-α silencing. We detected a very low level of cleaved caspase3-immunoreactive DRG neurons under naïve basal conditions (3.35±1.00%, **Figure 6A, B**). However, the number of cleaved caspase3-positive neurons in ipsilateral L5 DRG 14 days after SNT increased significantly in the LV-GFP group (16.52±1.56%); this SNT-induced increase in number of apoptotic cells was significantly suppressed by LV-TNF-shRNA3 treatment (to 9.09±1.14%) (**Figure 6A, B**).

### LV-TNF-shRNA3 reduces mRNA expression of neuropeptides, injury and inflammatory markers related to neuropathic pain

**Figure 7:**
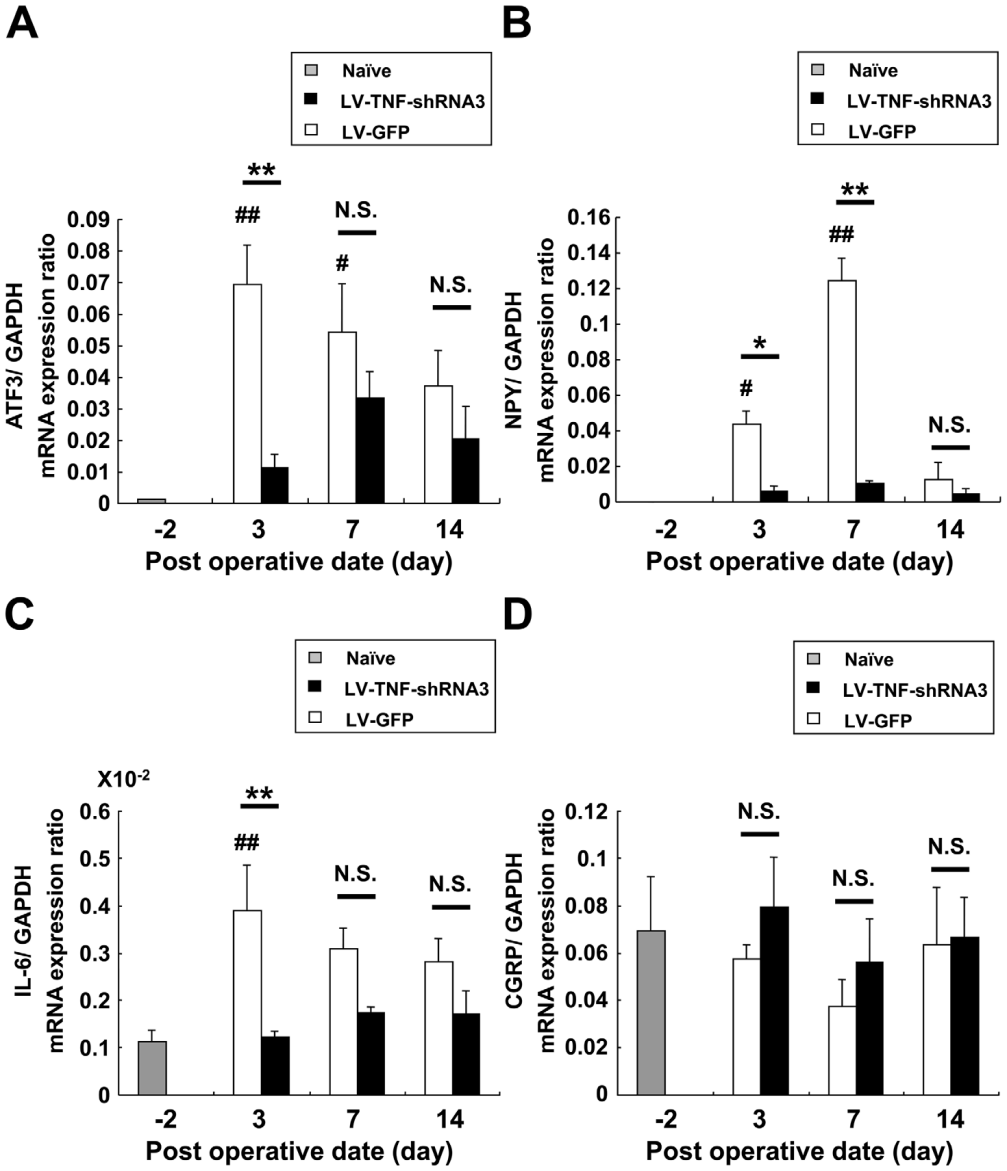
mRNA expression of neuropeptides, inflammatory and injury markers in SNT mice L5 DRG with treatment. ATF3 (**A**), NPY (**B**), IL-6 (**C**) and CGRP (**D**) mRNA expression were measured on day 3, 7 and 14 after SNT. The data are normalized to GAPDH. Gray bars - naïve DRG (on day −2); white bars - LV-GFP group; black bars - LV-TNF-shRNA3 group (n = 6–8 per group). Bars show means ± S.E. *p<0.05, **p<0.01, ^#^ *p*<0.05 compared with naïve, ^##^ *p*<0.01 compared with naïve. N.S. means “not significant”.

Many injury markers, cytokines and neuropeptides have been reported to be up-regulated in DRG neurons in mouse models of neuropathic pain. Thus, we measured the mRNA expression of the injury makers ATF3 and NPY, the inflammatory cytokine IL-6, and the neuropeptide CGRP in ipsilateral L5 DRG to evaluate the effect of TNF-α silencing. Both ATF3 and NPY mRNA levels increased markedly after SNT. The ATF3 mRNA peaked early at day 3, whereas NPY mRNA increased significantly at day 3 but peaked later at day 7 after SNT (**Figure 7A, B**). The peak ATF3 expression was significantly suppressed on day 3 and that of NPY was significantly suppressed on days 3 and 7 in the LV-TNF-RNA3 group compared with the LV-GFP group (**Figure 7A, B**). IL-6 mRNA expression was significantly elevated in L5 DRG by SNT in the LV-GFP group on day 3 (**Figure 7C**), and the level was significantly attenuated in the LV-TNF-shRNA3 group on day 3 (**Figure 7C**). We detected no significant change in the mRNA expression of CGRP, a neurotransmitter correlated with pain transmission (**Figure 7D**), and its level was not changed by LV-TNF-shRNA treatment (**Figure 7D**).

### LV-TNF-shRNA3 gene therapy for neuropathic pain in the SNT mouse model

**Figure 8:**
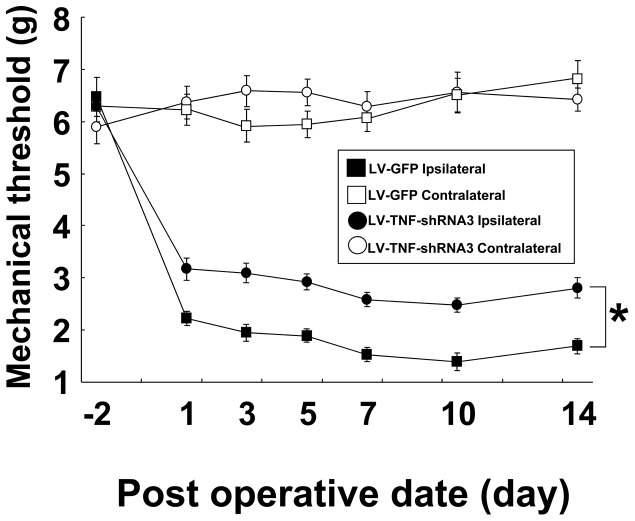
The effect of gene therapy with LV-TNF-shRNA3 for neuropathic pain in SNT mice. The threshold of mechanical allodynia was measured on the ipsilateral and contralateral sides in a neuropathic pain model for 14 days after SNT, in mice administered LV-TNF-shRNA3 or LV-GFP (as a control) (n = 6-7 per group). White circles - contralateral side of LV-TNF shRNA3 group; black circles - ipsilateral side of LV-TNF shRNA3 group; white squares - contralateral side of LV-GFP group; black squares - ipsilateral side of LV-GFP group. Data indicate means ± S.E. *p<0.01 between LV-TNF-shRNA3 ipsilateral and LV-GFP ipsilateral group.

While we analyzed the various biochemical and immunohistochemical parameters induced by SNT, we also monitored the efficacy of LV-TNF-shRNA3 or LV-GFP (control vector) treatment by measuring the mechanical threshold every other day till day 7 and again on day 10 and 14 after treatment (**Figure 8**). The mechanical threshold in the ipsilateral hind paw was significantly impaired in both groups from day 1 to day 14, but the impairment was significantly attenuated throughout this period in the LV-TNF-shRNA3 group compared with the LV-GFP (control vector) group. Therefore, the LV-TNF-shRNA3 treatment led to an improvement in an objective behavior measurement in neuropathic pain in SNT mice.

## Discussion

This study demonstrated that silencing of TNF-α expression with lentiviral vectors suppressed neuropathic pain induced by SNT. Lentiviral vectors targeted to DRG tissues decreased TNF-α expression in the affected (ipsilateral) DRG neurons. In addition, silencing of TNF-α expression in DRG resulted in the inhibition of neuronal cell death and apoptosis caused by SNT. Furthermore, injury markers and inflammatory cytokines that were up-regulated by SNT were greatly attenuated by lentiviral gene therapy. Therefore, gene therapy-mediated TNF-α silencing may be an efficacious treatment strategy for protecting DRG neurons and ameliorate neuropathic pain induced by nerve injury.

### Neuropathic pain and TNF-α

Neuropathic pain is caused by neuronal damage or dysfunction [1], [35]. Recent studies suggest that a communication between the immune and the nervous systems may underlie neuropathic pain [11]. TNF-α is a pro-inflammatory cytokine commonly produced in response to different types of disease, inflammation and tissue damage. It is a short-lived inflammatory mediator that plays a central role in initiating inflammatory reactions of the innate immune system, including the induction of other cytokine production, activation and expression of adhesion molecules, and stimulation of inflammatory cells in nerve injury [36]. Indeed, the direct application of TNF-α to the sciatic nerve and DRG leads to changes in the properties of neurons, such as ectopic firing in Aδ-, Aβ- and C-fibers [37], [38], and induces thermal hyperalgesia and mechanical allodynia [18], [39], [40]. Furthermore, TNF-α enhances TTX-R Na^+^ channels and increases membrane K^+^ ion conductance by a non-voltage-gated mechanism in DRG neurons [41], [42]. Therefore, TNF-α plays a pivotal role in multiple pathways involved in neuropathic pain and is clearly a treatment target for neuropathic pain. Here we found that silencing TNF-α in DRG partially suppressed mechanical allodynia though it did not produce a complete remission. Other cytokines, neurotrophic factors and chemokines such as IL-1β, nitric oxide, ATP, chemokine(C-C motif) ligand 2 and nerve growth factor, are involved in the pathogenesis of neuropathic pain, and they have direct actions as well as mediate crosstalks with TNF-α in nociceptive cascade in DRG neurons [43]. It is likely that the directed modulation of these molecules, in addition to TNF-α suppression, may be required for a more robust clinical remission of the neuropathic pain. Nonetheless, this study showed that gene therapy-mediated TNF-α-silencing is a useful strategy for the treatment of neuropathic pain.

### Association between TNF-α and apoptosis or inflammation in spinal nerve injury

Spinal nerve injury leads to activated caspase3 and increased apoptosis [34] in DRG neurons [44], [45]. TNF-α was known to be associated with apoptosis through the activation of caspase3 pathway [46]. In this study, TNF-α expression and caspase3 were elevated in DRG tissues 14 days after SNT, and we showed that TNF-α silencing in this setting suppressed caspase3 and increased the number of surviving DRG neurons. Therefore, silencing TNF-α not only suppresses neuropathic pain but also protects DRG neurons from apoptotic cell death after SNT, an effect that may have an impact on the functional maintenance and regeneration potential of sensory neurons.

IL-6 is up-regulated in DRG in response to peripheral nerve injury [47], [48]. A recent study suggested that TNF-α induces the production of IL-6, which contributes to neuropathic pain in DRG [49] and spinal cord in a spinal nerve transection model [47]–[49]. ATF3 and NPY are widely used as injury markers of axotomy. These two molecules are also up-regulated in DRG neurons in nerve injury models [50], [51], and are associated with phenotypic changes [52] in DRG neurons such as ion channel excitability and responses against neuropeptides [53], [54]. Here, we showed that mRNA expression of ATF3, NPY and IL-6 is elevated in the early phase after SNT but can be suppressed by TNF-α silencing, indicating that TNF-α is likely up-stream of the immune cascade during nerve injury. The multiple downstream effects of TNF-α silencing protect DRG neurons against inflammation induced by nerve injury by suppressing of ATF3, NPY and IL-6 expression, resulting in the reduction of apoptosis and suppression of neuropathic pain.

### Benefits of lentiviral vector-mediated silencing of TNF-α

DRG-targeted gene delivery is a promising therapeutic option for the treatment of neuropathic pain. A number of viral gene vectors are available for gene delivery, including adenoviral (Ad) vectors, adeno-associated viral (AAV) vectors, and lentiviral vectors [55]. The selection of the appropriate viral vectors and route of delivery are important issues that must be considered.

In general, Ad vectors have high transduction efficiency in most cells with the coxsackie virus and adenovirus receptor (CAR) compared to AAV and lentiviral vectors after attachment to the target cells [56]–[58]. However, Ad vectors show relatively low affinity for mature neuronal cells with rare CAR [56], [59]. In using AAV vectors for neural tissue transduction, several serotypes of AAV vectors are known to show tropism to neurons [55], [60]. In lentiviral vectors, the tropism to the neurons is acquired by the specific envelop protein [55], [61]–[63]. In this study, we used lentiviral vectors pseudotyped with vesiculo-stomatitis virus glycoprotein for gene therapy of neuropathic pain because this glycoprotein gives the letiviral vectors both the potential of tropism for neuronal cells and an ability to infect neuronal cells by axonal transport [63].

It is also important to consider the toxicity of various viral vectors. Ad vectors have been shown to induce a potent immune response against the capsid, double-stranded DNA genome, viral proteins expressed from the vector backbone, or incorporated transgenes, a limitation for *in vivo* gene therapy [64]. In contrast, AAV and lentiviral vectors generally elicit a relativelymild immune response as compared to Ad vectors [55], [64]. Using lentiviral vectors might lead to a relatively mild immune response, when used *in vivo* in rodents [55], [56], [62].

For delivering transgenes by viral vectors to the DRG *in vivo*, it was reported mainly by three different approaches, intrathecally, direct injection into sciatic nerve or into the DRG itself [55], [60]–[62], [65], [66]. A prior study using AAV vectors suggested that the direct injection of the vectors into the DRG or sciatic nerve could provide stronger gene expression in the DRG neurons than injection into subcutaneous tissue or subarachnoid space [65]. Moreover, direct injection of Ad vector and AAV vector into DRG was also reported to be vastly superior to intraneural injection into sciatic nerve [66]. Lentiviral vectors have been delivered to either directly into DRG or indirectly via the sciatic nerve, which also ended up in the DRG [61], [62]. In general, direct DRG injection appears to be the most effective and widely used approach. We note, however, that direct injection to DRG may induce additional tissue damage. Especially for the treatment of neuropathic pain, it is important to avoid further damage of the sensory nerve system, which by itself may induce neuropathic pain [67]. Here, we selected injection onto injury site of spinal nerve as a promising approach.

Unexpectedly, we found that spinal nerve injection led to high level transduction of the DRG. It was much higher than that reported in a previous study that utilized direct injection of lentiviral vectors into DRG in rats [62]. The administration method of injection onto injury site of spinal nerve seems to produce results from three different delivery methods, combining the effects of intrathecal injection, direct sciatic nerve or DRG injection. In fact, we demonstrated that GFP expression and silencing of TNF-α occurred in the ipsilateral as well as contralateral DRGs. The lentiviral vector might have spread through the cerebrospinal fluid to the contralateral DRG. This phenomenon is consistent with the expression pattern as transgene was found to be diffusely expressed at many spinal levels of DRG after intrathecal injection. At the same time, lentiviral vectors may be delivered to the DRG neurons by axonal transport because lentiviral vectors were injected onto spinal nerve in a manner similar to that following sciatic nerve injection as previously reports [56]. In our results, transgene was expressed diffusely in DRG neurons, which was different from the expression pattern immediately adjacent to the injection site following direct injection into DRG. Finally, it is possible that lentiviral vectors may access the ipsilateral DRG by direct infiltration. Here, we showed that lentiviral vectors injected onto the injury site of the spinal nerves led to high efficiency transduction of DRGs, constituting a promising approach for delivering transgenes to the DRG, although further studies should be needed to clarify the details of mechanism of their high efficiency.

### Clinical relevance of lentiviral vector-mediated silencing of TNF-α

Clinical reports suggest that anti-TNF-α drugs, such as etanercept [7], [28], [29] and infliximab [25], [30], have the potential to relieve neuropathic pain caused by sciatica or disc herniation. In addition, HIV chemotherapy may lead to peripheral neuropathy caused by the massive release of TNF-α in serum and TNF-α therapy for cancer was shown to cause peripheral neuropathy [68], [69]. Thus, silencing TNF-α, an initiator of the immune cascade, could be a therapeutic target for different types of neuropathic pain. However, anti-TNF-α drugs administered systemically is associated with many side effects, e.g., general immunosuppression in patients. The strategy used in this study addresses this problem because the gene was selectively delivered locally to the injury site. There are many potential clinical applications for the treatment method developed in this study. On the other hand, we must also contend with potential lentiviral toxicity. Our data showed the TNF-α mRNA expression ratio increased slightly in the LV-GFP group compared with SNT only (no infection), which might be have been induced by the gene delivery of lentiviral vectors, GFP, and/or undesired proteins remaining in the preparation of lentiviral vectors after the purification processes.

Considering the gene therapy of sensory neuronopathy, we previously reported the intrathecal administration of DRG-targeting fiber-modified helper-dependent adenovirus (HDAd) vectors [70]. The fiber-modified vectors display high affinities for neurons compared to the wild type Ad vectors. However, injection onto spinal nerve of lentiviral vectors showed quite high transduction efficiency to DRG neurons, which seems to be as good as, if not better than, the fiber-modified HDAd vectors. As some psuedtyped lentiviral vectors generally have high tropism to neurons compared with adenoviral vectors, local injection of lentiviral vectors showed high performance without recombination like as the modification of fiber protein. Moreover, the target gene delivered by lentiviral vectors can be integrated into the nuclei of neuronal cells leading to stable and long-term expression of therapeutic genes [71]. It obviates the necessity for repeated injections which might lead to an immune response. Therefore, we believe that lentiviral vectors may be the optimal vector for long-term therapy and may be ideal for the treatment of sensory neuronopathies or chronic neuropathic pain as this study.

In conclusion, we presented a strategy to relieve neuropathic pain using lentiviral shRNA vectors. Our results support previous evidence for TNF-α in the pathogenesis of neuropathic pain. In addition, we showed that TNF-α silencing by lentiviral shRNA vectors protects injured DRG neurons from apoptosis. Silencing TNF-α by lentiviral vector-mediated gene transfer targeted to DRGs with injection onto injury site of spinal nerve may provide a novel therapeutic alternative for the treatment of neuropathic pain.
